# Genome sequencing and comparative genomics reveal insights into pathogenicity and evolution of *Fusarium zanthoxyli*, the causal agent of stem canker in prickly ash

**DOI:** 10.1186/s12864-024-10424-w

**Published:** 2024-05-21

**Authors:** Zhao Ruan, Jiahui Jiao, Junchi Zhao, Jiaxue Liu, Chaoqiong Liang, Xia Yang, Yan Sun, Guanghui Tang, Peiqin Li

**Affiliations:** 1 Key Laboratory of National Forestry and Grassland Administration on Management of Western Forest Bio- Disaster, College of Forestry, Northwest A&F University, Yangling, Shaanxi 712100 People’s Republic of China; 2 Shaanxi Academy of Forestry, Xi’an, Shaanxi 710082 People’s Republic of China

**Keywords:** Genome sequencing, *Fusarium zanthoxyli*, Comparative genomics, Phylogenomic evolution, Pathogenicity-related factors

## Abstract

**Background:**

*Fusarium zanthoxyli* is a destructive pathogen causing stem canker in prickly ash, an ecologically and economically important forest tree. However, the genome lack of *F. zanthoxyli* has hindered research on its interaction with prickly ash and the development of precise control strategies for stem canker.

**Results:**

In this study, we sequenced and annotated a relatively high-quality genome of *F. zanthoxyli* with a size of 43.39 Mb, encoding 11,316 putative genes. Pathogenicity-related factors are predicted, comprising 495 CAZymes, 217 effectors, 156 CYP450s, and 202 enzymes associated with secondary metabolism. Besides, a comparative genomics analysis revealed *Fusarium* and *Colletotrichum* diverged from a shared ancestor approximately 141.1 ~ 88.4 million years ago (MYA). Additionally, a phylogenomic investigation of 12 different phytopathogens within *Fusarium* indicated that *F. zanthoxyli* originated approximately 34.6 ~ 26.9 MYA, and events of gene expansion and contraction within them were also unveiled. Finally, utilizing conserved domain prediction, the results revealed that among the 59 unique genes, the most enriched domains were PnbA and ULP1. Among the 783 expanded genes, the most enriched domains were PKc_like kinases and those belonging to the APH_ChoK_Like family.

**Conclusion:**

This study sheds light on the genetic basis of *F. zanthoxyli*’s pathogenicity and evolution which provides valuable information for future research on its molecular interactions with prickly ash and the development of effective strategies to combat stem canker.

**Supplementary Information:**

The online version contains supplementary material available at 10.1186/s12864-024-10424-w.

## Background

**Fig. 1:**
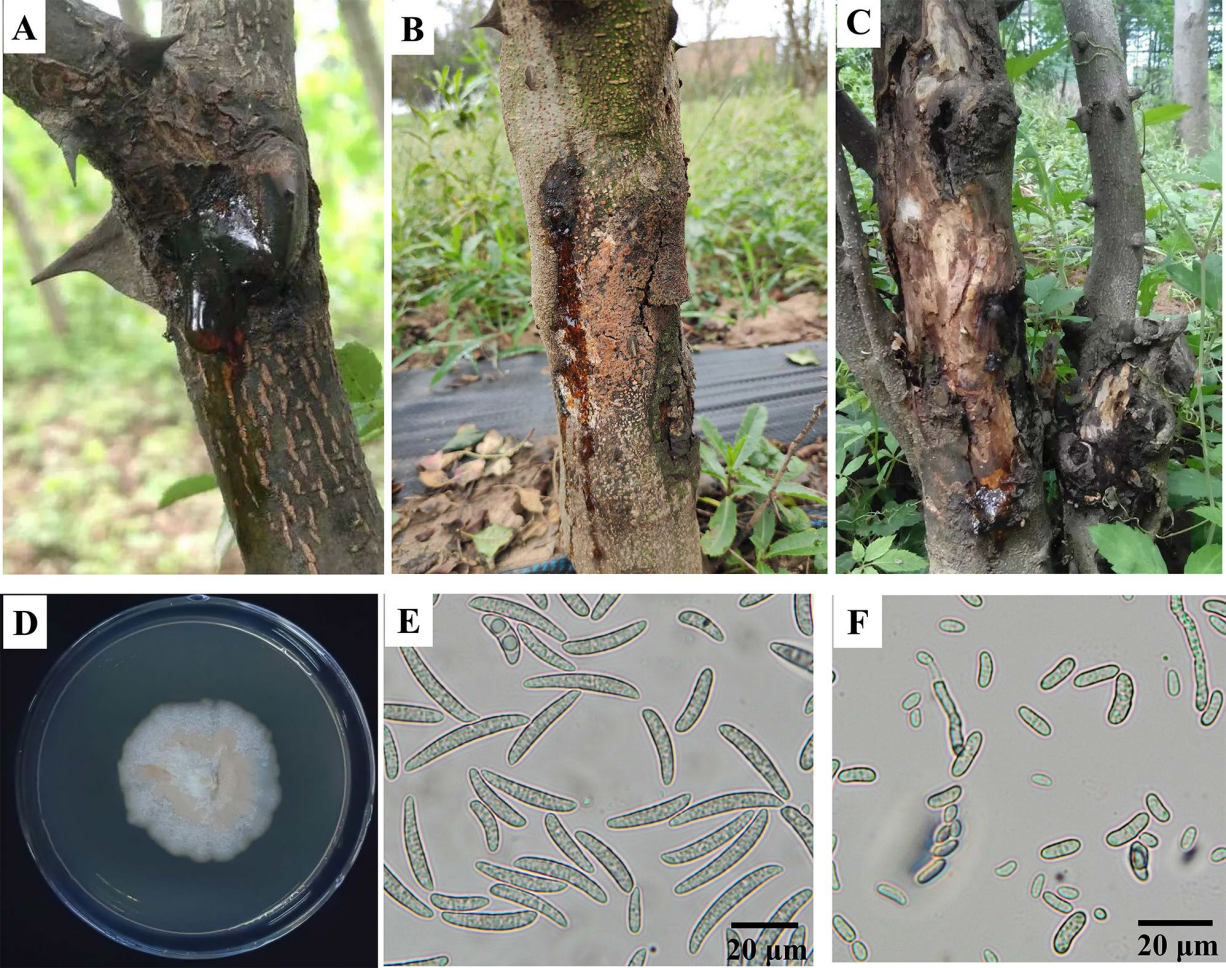
Symptoms of *Z. bungeanum* stem canker and morphological features of *F. zanthoxyli*. (**A**) Early canker. (**B**) Later canker. (**C**) Long-term stem canker in *Z. bungeanum*. (**D**) Fourteen-day colony culture of *F. zanthoxyli*. (**E**) Macroconidia. (**F**) Microconidia.

*Zanthoxylum bungeanum*, commonly known as prickly ash in China, is a tree species of great economic importance within the Rutaceae. It is widely distributed in various Asian countries, including China, Japan, India, and Korea [1]. The pericarp of *Z. bungeanum* fruit is renowned for its delicate and addictive pungency, and has a long history of use as a culinary spice and condiment in the catering and food industry [2]. Given its immense economic and medicinal value, the cultivation of *Z. bungeanum* has become a crucial commercial venture for farmers in China. Currently, *Z. bungeanum* is extensively planted in dry and mountainous areas across several provinces in China due to its fast growth and adaptability to adverse soil and climatic conditions [3]. However, *Z. bungeanum* is susceptible to various plant diseases during its growth period of which is particularly prevalent. Stem canker manifests as branch and stem cankers, dieback, and occasional tree death, and is often accompanied by gummosis [4]. Stem canker not only severely affects the growth of *Z. bungeanum* but also leads to a significant decrease in quality and yield. The pathogens responsible for stem canker in *Z. bungeanum* have been identified as two novel *Fusarium* species: *F. zanthoxyli* and *F. continuum*. These two species exhibit a close genetic affiliation with *F. torreyae*, the pathogen causing stem canker in *Torreya taxifolia* trees in Florida, USA [5]. Thus, these three pathogens collectively form a new phylogenetic branch within the *Fusarium* genus known as the *Fusarium torreyae* species complex (FTOSC). Currently, there is limited knowledge regarding the genomic information of the pathogens within FTOSC. In *Z. bungeanum-*producing provinces in China, *F. zanthoxyli* exhibits a broader distribution compared to *F. continuum.* Detailedly, *F. zanthoxyli* is widely distributed in Gansu, Shanxi and Shaanxi provinces, while *F. continuum* is mainly found in Shandong province [5]. The typical symptom of *Z. bungeanum* stem canker caused by *F. zanthoxyli* is presented in Fig. 1. In the early stage of the growing season, obvious gummosis can be observed (Fig. 1A), while in the later stage, abundant pinkish-orange sporodochia and conidia are produced on the surface of necrotic bark (Fig. 1B). Stem canker can repeatedly damage *Z. bungeanum* over several years, resulting in bark slipping and xylem necrosis (Fig. 1C). The colony growth of *F. zanthoxyli* on potato dextrose agar (PDA) medium showed fewer aerial hyphae and a slow growth rate with a value of 3.1 mm/d (Fig. 1D). Additionally, *F. zanthoxyli* is capable of producing two types of conidia: macroconidia (Fig. 1E) and microconidia (Fig. 1F).

To date, no other plant hosts of *F. zanthoxyli* have been reported besides *Z. bungeanum*, suggesting a specific interaction between *F. zanthoxyli* and *Z. bungeanum*. Deciphering this kinds of interaction between pathogens and plants can provide significant guidance for the precise control of plant disease. Understanding the pathogenicity of pathogens is pivotal to the further researches on the interplay between plants and pathogens. Obtaining the genome information of a plant pathogen and its associated annotated genes is a prerequisite for gaining insights into its mechanisms of pathogenicity, host-specific mediator, pathogen biochemistry, physiology and adaptation to ecological niches. Therefore, it is extremely urgent to sequence the genome of *F. zanthoxyli* and comprehensively demonstrate its genomic features and phylogenetic evolution.

The advent of the genomic era has revolutionized our understanding of fungal plant pathogens. Following the landmark publication of the *Magnaporthe oryzae* genome [6], there has been an exponential increase in the sequencing of genomes of fungal plant pathogens. *Fusarium*, a widely distributed genus of filamentous Ascomycota fungi (Sordariomycetes: Hypocreales: Nectriaceae), encompasses numerous plant pathogens that have a major agricultural impact and are known for their ability to produce toxins [7]. Collectively, *Fusarium* species can cause wilt, blight, rot, and canker in *diverse* horticultural, field, ornamental, and forest crops in both agricultural and natural ecosystems [7]. It has been reported that the *Fusarium* genus comprises at least 450 phylogenetic species within 23 divergent species complexes [8]. Currently, over 200 species of *Fusarium* fungi have been subject to genome sequencing, such as *F. graminearum* (GCF_000240135.3), *F. oxysporum* (GCF_000271745.1), *F. solani* (GCF_020744495.1), *F. vanettenii* (GCA_020744135.1), and others. These genome sequences have provided valuable information for the accurate analysis of *F. zanthoxyli*. Genomic technology has been widely utilized to uncover pathogenic genes in fungal pathogens, including critical components such as CAZymes, effectors, and secondary metabolites, etc [9]. Among these key players, CAZymes play a critical role in the synthesis, modification, and degradation of polysaccharides, which are essential for breaking through the host cell wall during the infection process [10]. Effector proteins, another vital class of virulence factors, show remarkable prowess in promoting infection and colonization by targeting plant defense components, signaling, and metabolic pathways [11, 12]. Additionally, pathogenic fungi can produce numerous secondary metabolites (SMs) that are not essential for their survival but provide advantages in fungal-host interactions under natural conditions [13]. In essence, the genomic revolution has facilitated extensive exploration of various plant pathogenic fungi and has opened up new horizons in our understanding of fungal pathogenicity.

Analyzing a single genome provides insights into its gene characteristics and biological processes, while comparative analyses of two or more genomes allow for the identification of conserved and divergent features [14]. Therefore, comparative genomics has been widely utilized to investigate similarities and differences among species, genera, higher order clades, and intraspecific variability [15]. Population comparative genomics studies have revealed the historical and ongoing recombination in the *Fusarium oxysporum* species complex (FOSC) [16]. Comparative genomics analyses also enable the identification of expanded and contracted gene families in the target species, which may play key roles in its evolution, adaptability and pathogenicity [17]. For example, through the analysis of genome sequences from seventeen species belonging to six different genera of Botryosphaeriaceae, the pathogenicity of these pathogens may be associated with the expansion of gene families involved in secreted CAZymes, secondary metabolism, and transporters [18]. Conversely, rapid contractions of gene families have been linked to biotrophy and shifts in host preferences [19]. Notably, obligate biotrophic phytopathogens exhibit significant reductions in gene families responsible for CAZymes and proteases compared to hemibiotrophic and necrotrophic phytopathogens. For instance, comparative analysis has revealed substantial gene contractions in CAZymes, transporters, primary metabolism, and secondary metabolism in obligate biotrophic phytopathogens such as *Blumeria graminis*, *Erysiphe pisi*, and *Golovinomyces orontii* when compared to hemibiotrophic fungal pathogens like *Colletotrichum higginsianum* and *Magnaporthe oryzae*, as well as necrotrophic fungal pathogens like *Sclerotinia sclerotiorum* [20]. This phenomenon is likely attributed to their adaptation to the obligate biotrophic lifestyle. In addition, a comprehensive phylogenomic analysis of 45 fungal genomes from seven families in Hypocreales demonstrated a significant reduction in carbohydrate-degrading CAZymes during the transition of lifestyle from plant pathogens to other forms such as animal pathogens, mycoparasites, or endophytes [21]. This finding highlights the versatility of these gene families in different ecological contexts. In essence, comparative genomics provides a robust platform for unraveling the complex genetics underpinning plant-pathogen interactions. It offers insights into the evolution and adaptation of gene families within diverse ecological conditions and lifestyles, shedding light on the dynamic interplay between pathogens and their hosts.

In this study, we conducted a comprehensive analysis of the genome of *F. zanthoxyli*, including genome sequencing, assembly, and annotation to generate a relatively high-quality genomic dataset. We mined and characterized the pathogenicity factors in *F. zanthoxyli*, such as CAZymes, effectors, and enzymes involved in secondary metabolite production. To gain insights into the paleohistory of *F. zanthoxyli*, we conducted a comparative genomic investigation by analyzing the genomes of *F. zanthoxyli* and 25 other fungal species of Ascomycota and Basidiomycota. Additionally, we explored the phylogenomic evolutionary relationships between *F. zanthoxyli* and other *Fusarium* species. This analysis involved demonstrating affiliations, estimating divergence times, gene expansion and contraction events, as well as identifying unique and common genes. Furthermore, we analyzed the co-linearity and selective evolutionary pressure between *F. zanthoxyli* and four typical phytopathogenic *Fusarium* species. Overall, this study contributes to an improved understanding of the interaction mechanisms between *F. zanthoxyli* and *Z. bungeanum* while enriching our knowledge of the molecular genetics of plant pathogenic *Fusarium* species. The findings from this study will provide crucial guidance for precise prevention and control strategies against *Z. bungeanum* stem canker.

## Materials and methods

### Fungal strain

The pathogen of *Z. bungeanum* stem canker, *F. zanthoxyli* Fz001, was kindly provided by Professor Zhimin Cao (the former leader of our research team, Forest Pathology Lab, Forestry College, Northwest A&F University) [5]. *F. zanthoxyli* stored at -80 °C in paraffin wax was inoculated on PDA medium and cultured for 7 days at 25 °C. Thereafter, the mycelial plug of *F. zanthoxyli* was reinoculated on fresh PDA medium at 25 °C for 7 days, which was repeated twice to revive the pathogen. Subsequently, the mycelium of Fz001 was collected and used as the material for DNA extraction.

### Plant material and growth conditions

The cultivar of *Z. bungeanum* highly susceptible to *F. zanthoxyli*, Fengxian Dahongpao (FD) [4], was used in this study. Two-year-old seedlings of FD were graciously provided by a prickly ash orchard (the Research Center for Engineering and Technology of *Zanthoxylum*, National Forestry Administration, located in Fengxian County, Shaanxi Province, China, 33°59′N, 106°39′E). These seedlings were cultivated in an environmentally controlled greenhouse at Northwest A&F University (Yangling, China) under a temperature of 25 ± 2 °C, a relative humidity of 75%, and a photoperiod of 12 h light/12 h dark with a light intensity of 2000 Lx.

### Library construction, sequencing and assembly

The genome of *F. zanthoxyli* was sequenced using Single Molecule Real-Time (SMRT) technology [22] by the Beijing Novogene Bioinformatics Technology Co., Ltd (Beijing, China). Genomic DNA of *F. zanthoxyli* was extracted using the sodium dodecyl sulfate method. Libraries for SMRT sequencing were constructed with an insert size of 20 kb using the SMRTbell™ Template Kit, and then sequenced using the long-reads PacBio Sequel platform. The DNA quality was assessed by agarose gel electrophoresis and quantified with a Qubit® 2.0 fluorometer (Thermo Fisher Scientific, Waltham, MA, USA). Additionally, Illumina sequencing was conducted using the NEBNext® Ultra™ DNA Library Prep Kit and the Illumina NovaSeq PE150 platform to survey the genome. The Illumina data were utilized for assembly error correction, where low-quality reads were filtered using SMRT Link 5.0.1, and the long reads were selected as seed sequences for alignment with shorter reads to improve accuracy and generate a contiguous assembly. The regions with assembly errors were further polished using the arrow algorithm in the Varian Caller module of SMRT Link 5.0.1. Furthermore, by determining the GC content of the assembled sequence and the coverage depth of reads, the GC bias and repeated sequences of the genome were estimated. Additionally, a BUSCO (Benchmarking Universal Single-Copy Orthologs) testing was conducted to evaluate the integrity of the assembled genome of *F. zanthoxyli.*

### Genome component prediction and gene annotation

Whole-genome gene prediction for *F. zanthoxyli* was performed with AUGUSTUS and homology-based gene prediction was conducted with GeneWise 2.4.1 using the homologous protein sequence of *F. graminearum* PH-1(GCA_900044135) as the reference sequence. Dispersed repeats sequences (DRs) were predicted with Repeat Masker, while tandem repeats sequence (TRs) were predicted using Tandem Repeats Finder. Transfer RNA (tRNA) genes, ribosomal RNA (rRNA) genes, and small RNA (sRNA) genes were respectively predicted by tRNAscan-SE, rRNAmmer, and a BLAST search of the Pfam database.

To functionally annotate the predicted genes of *F. zanthoxyli*, the predicted protein sequences were subjected to Diamond analyses in the following biological information databases, namely Gene Ontology (GO), Kyoto Encyclopedia of Genes and Genomes (KEGG), euKaryotic Orthologous Groups (KOG), Non-Redundant Protein Databases (NR), Transporter Classification Database (TCDB), Pfam, Swiss-Prot database, Carbohydrate-Active Enzymes Database (CAZy), and Cytochrome P450 database (CYP450) (with E-value less than 1e − 5 and minimal alignment length percentage greater than 40%). The method used for the prediction of the *F. zanthoxyli* secretome and effectors was modified from previous studies [23]. The secretory proteins were predicted using the online tools of SignalP (v.6.0), WoLF PSORT, TargetP (v.2.0), Deep TMHMM, and big-PI Predictor. Finally, based on the results obtained, EffectorP 3.0, a predictive tool for effectors of plant pathogenic fungi and oomycetes, was used to predict candidate effector proteins of *F. zanthoxyli*. The genes associated with the pathogenicity of *F. zanthoxyli* were predicted using the Pathogen-Host Interaction Database (PHI) and Database of Fungal Virulence Factors (DFVF). The secondary metabolism gene clusters were predicted using the online tool antiSMASH 2.0.2.

### Comparative genomics analysis

Genomic sequences of 26 fungal species, including *F. zanthoxyli*, from eight classes of Ascomycota and Basidiomycota (Table S1) were obtained from the NCBI and JGI databases. Orthofinder (v.1.1.4) [24] was then utilized to identify orthologous gene clusters across all 26 fungal species. Multiple sequence alignments of single-copy orthologous genes were performed using MAFFT [25], and poorly aligned regions were removed using Gblocks (v.0.91b) [26]. A phylogenomic tree was constructed using IQ-TREE (v.1.6.12) based on maximum likelihood inference [27] to reveal the evolutionary relationships of *F. zanthoxyli*. To estimate divergence times, molecular clock models were applied by calibrating the molecular clock using fossil records or known divergence events. The MCMCTree program in the PAML package (v.4.9) [28] was used to incorporate multiple calibration points and account for uncertainties in divergence time estimation. Fossil records were obtained from the TimeTree database. Gene expansion and contraction events were also analyzed using CAFÉ (v.2.1) [29]. The birth and death rates of orthologous gene families across all 26 species were estimated to identify significantly expanded or contracted gene families. In addition, further assessment of the evolutionary relationships between *F. zanthoxyli* and other *Fusarium* species complexes was conducted (Table S2). The phylogenomic evolution, differences in divergence times, and gene expansion and contraction events were analyzed using the same methods as described above.

To better understand the differences in the genetic organization and evolution of *F. zanthoxyli* and four common phytopathogenic *Fusarium* species, namely, *F. solani*, *F. graminearum*, *F. oxysporum* and *F. sporotrichioides*, genomic co-linearity analyses were conducted using TBtools [30]. The Ka/Ks ratios for single-copy orthologs among these species were further evaluated with TBtools. Genes were categorized based on their Ka/Ks ratio values. Genes with a Ka/Ks ratio less than 1 were considered to have undergone purifying selection, indicating that natural selection has favored the preservation of these genes [31]. Genes with a Ka/Ks ratio equal to 1 were considered to have undergone neutral selection, suggesting that these genes have evolved without significant selective pressure. Lastly, genes with a Ka/Ks ratio greater than 1 were considered to have evolved under positive selection, indicating that these genes have experienced adaptive changes driven by positive selective forces.

### Conserved domain prediction of unique and expanded genes in ***F. zanthoxyli***

The unique and expanded genes in *F. zanthoxyli* identified through the comparative genomics across the 12 *Fusarium* species (Table S2) were subjected to conserved domain analyses using NCBI-CDD to predict the potential functions of these genes [32]. The conserved domains were identified with an E-value cutoff of 0.01. TBtools [30] was used to visualize the conserved motif, domain and gene architectures.

## Results

### Genome sequencing, assembly and annotation of ***F. zanthoxyli***

**Fig. 2:**
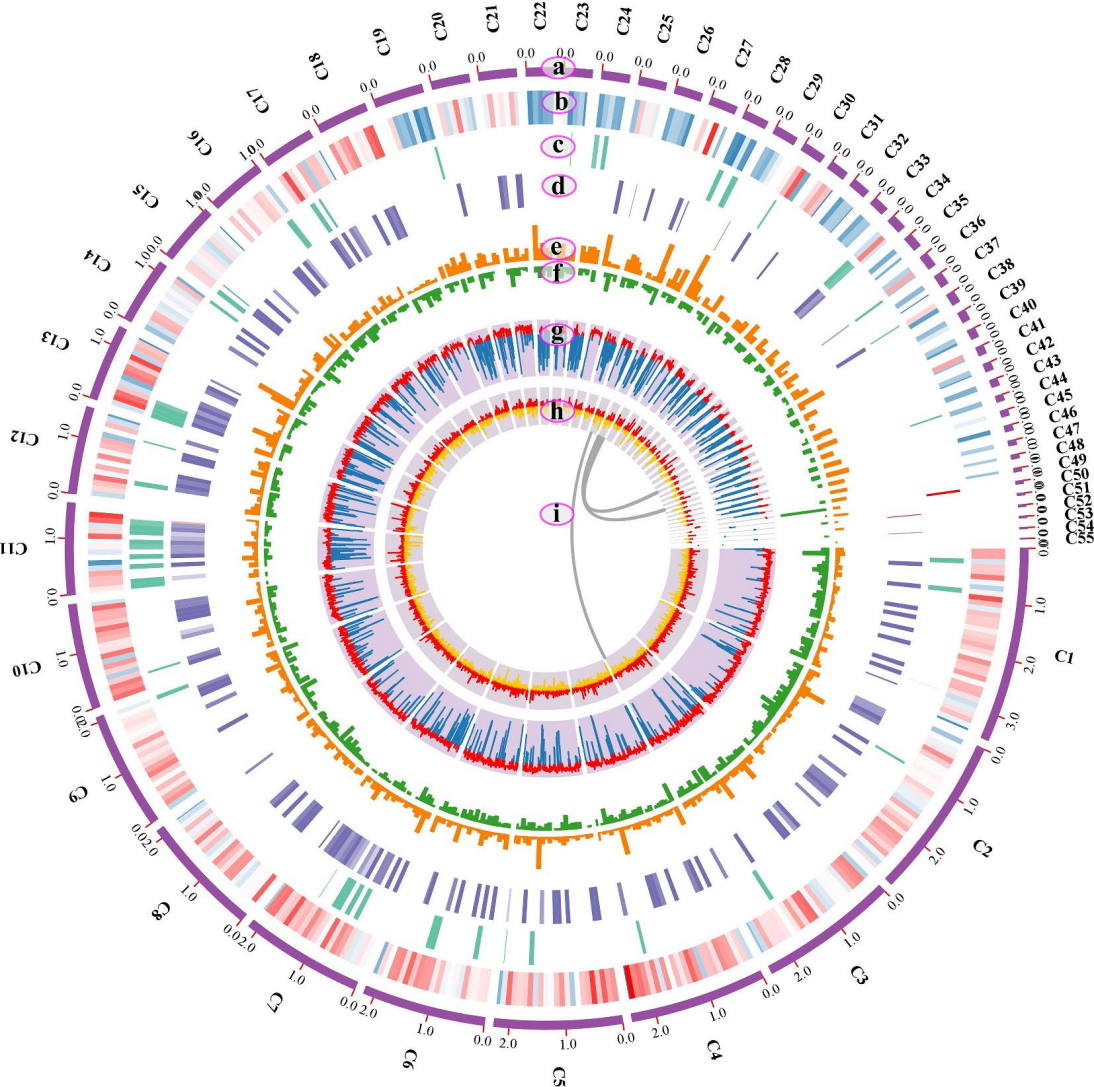
An optical schematic representation of the genomic features of *F. zanthoxyli*. Track a: Arrangement of contigs. The scale for the contigs (outer bars) is in megabases. Track b: Distribution of protein-coding gene density in the contigs. Red color represents a high density, while blue color represents a low density. Track c: Distribution of rRNA in the genome. Higher density is represented by darker cyan color. Track d: Distribution of tRNA in the genome. Higher density is represented by darker purple color. Track e: Bar plot of DRs density in the genome; Track f: Bar plot of TRs density in the genome. Track g: Line plot showing the distribution of GC content in the genome. The red color indicates regions with a higher GC percentage than the average, while the blue color indicates regions with a lower GC percentage than the average. Track h: Distribution of GC-skew in the genome. Red color represents a positive GC-skew, indicating an excess of guanine over cytosine, while yellow color represents a negative GC-skew, indicating an excess of cytosine over guanine. Track i: Schematic presentation of synteny relationships in the genome

A total of 959,145 high-quality clean reads containing 7.32 Gb clean data with an average length of 7.63 kb were obtained after quality control, which were assembled into 55 polished contigs (Table 1). The assembly quality of the Fz001 genome was also evaluated by graphically representing the correlation between the sample GC content and sequencing depth (Fig. S1). A sequencing depth of 168× was achieved for the genome of Fz001. From these contigs, a 43.39 Mb genome of *F. zanthoxyli* was generated, with an N50 contig size of 2.09 Mb and a GC content of 45.16%. In additionally, a well-constructed optical circular genome map of *F. zanthoxyli* was obtained (Fig. 2). Through a combination of *de novo* gene prediction and sequence similarity comparison with multiple functional databases, a total of 11,316 putative protein-coding genes were annotated in the genome of *F. zanthoxyli* (Table S3). These genes had an average length of 1.53 kb and a GC content of 52.97%, and accounted for 39.82% of the *F. zanthoxyli* genome. Furthermore, we determined that the intergenic region length in the *F. zanthoxyli* genome was 26.11 Mb with a GC content of 40.01%, constituting for 60.18% of the total genome (Table 1). According to BUSCO testing, the calculated ratio of C was found to be high at 99.2%, while the ratio of D was low at 0.1% (Fig. S2). Previous studies have reported that when the ratio of C exceeds 90% and the ratio of D is below 10%, it signifies the integrity and reliability of the assembled genome [33]. Thus, these results indicate that the assembled genome of *F. zanthoxyli* in our research is both integral and reliable. Meanwhile, 12,541 TRs were predicted with a total length of 567,378 bp, 10,681 minisatellite DNA were predicted with a total length of 451,644 bp, and 932 microsatellite DNA were predicted with a total length of 38,362 bp (Table 2). Furthermore, 279 tRNAs, 64 5s rRNAs, 5 18s rRNAs, 7 28s rRNAs, 2 sRNAs and 21 snRNAs were predicted in the genome of *F. zanthoxyli*. In the genome of *F. zanthoxyli*, a total of 2,491 repetitive elements known as direct repeats (DRs) were predicted .

**Table 1:** Features of the assembled contigs and genome of *F. zanthoxyli*

Contigs and genome parameters	Features
**Contigs**	
Total number	55 contigs
Max Length	3.46 Mb
N50 Length	2.09 Mb
G + C content	45.16%
**Genome**	
Genome size	43.39 Mb
Number of protein-coding genes	11,316
Gene total length	17.28 Mb
Gene average length	1.53 Kb
Gene length / Genome	39.82%
GC content in gene region	52.97%
Intergenic region length	26.11 Mb
GC content in Intergenic region	40.01%
Intergenic length/Genome	60.18%

**Table 2:** Statistical results for DRs, TRs and ncRNAs in the genome of Fz001

Types		Number	Total Length (bp)	In Genome (%)
**DRs**	LTR	1,351	265,199	0.6112
	DNAT	537	69,280	0.1597
	LINE	514	41,370	0.0953
	SINE	42	2,575	0.0059
	RC	29	2,051	0.0047
	Unknown	18	1,321	0.003
	Total	2,491	373,333	0.8604
**TRs**	TR	12,541	567,378	1.3076
	Minisatellite DNA	10,681	451,644	1.0408
	Microsatellite DNA	932	38,362	0.0884
**ncRNAs**	tRNA	279	24,043	0.0554
	5s rRNA	64	7,442	0.0172
	18s rRNA	5	9,011	0.0207
	28s rRNA	7	31,020	0.0715
	sRNA	2	470	0.0011
	snRNA	21	3,273	0.0075

The length distribution of the 11,316 putative protein-coding genes was analyzed (Fig. S3). It was observed that 3,762 genes had a length of less than 1,000 bp, 5,061 genes were between 1,000 bp and 2,000 bp, and 2,493 genes were longer than 2,000 bp. These putative protein-coding genes were annotated using various public databases and bioinformatics software. Among the annotations, specific information was identified for 10,631 genes in the NR database, 10,378 genes in the KEGG database, 7,126 genes in the GO database, 7,126 genes in the Pfam database, 2,412 genes in the KOG database, 3,311 genes in the SwissProt database (Table S4). Additionally, annotations were found for 577 genes in the TCDB database and for 1,847 genes in the PHI database. Furthermore, specific annotation information was identified for 472 genes in the DFVF database and for 156 and 495 genes in the CYP450 and CAZy databases respectively (Table S4).

The genes annotated in the NR database, which accounted for approximately 93.95% of the total predicted genes in *F. zanthoxyli*, were subjected to alignment against other species (Fig. S4). The results revealed that the majority of these aligned genes showed homology to *Fusarium* species. In the KEGG database, a total of 10,378 genes were annotated, 8,979 of which were found to be enriched in 382 KEGG pathways (Fig. S5). Furthermore, 7,126 protein-coding genes underwent GO analyses and were functionally assigned to three classes: “molecular function” (8,402 terms), “cellular component” (8,558 terms), and “biological process” (13,814 terms) (Fig. S6).

A total of 7,126 genes in *F. zanthoxyli* were annotated in the Pfam database, and these genes were further grouped into 361 clans (Table S5). Through NCBI KOG mapping, a total of 2,412 genes in *F. zanthoxyli* were assigned to 25 different KOG categories (Fig. S7). Among these categories, “general function prediction only” had the highest number of genes, followed by “posttranslational modification, protein turnover, chaperones”, “amino acid transport and metabolism”, “translation, ribosomal structure and biogenesis”, and “energy production and conversion”. In the case of *F. zanthoxyli*, a total of 577 genes were annotated in TCDB, accounting for 5.10% of the putative protein-coding genes. These genes were categorized into different transporter types based on their functions. Among these annotated genes, the highest number belonged to electrochemical potential-driven transporters. This was followed by primary active transporters, channels/pores, incompletely characterized transport systems, accessory factors involved in transport, group transporters, and transmembrane electron carriers (Fig. S8). These findings provide insights into the potential transport mechanisms and roles of these genes in *F. zanthoxyli’*s pathogenicity.

### Prediction of potential pathogenic factors in the genome of ***F. zanthoxyli***

**Fig. 3:**
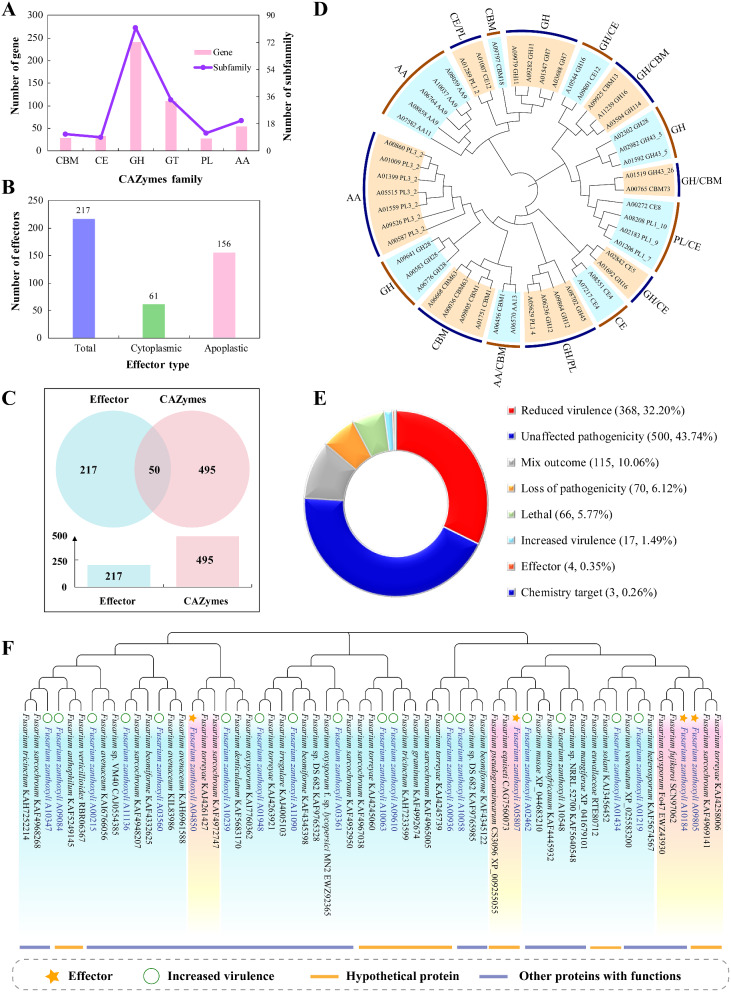
Comprehensive analyses of effectors, CAZymes and PHI proteins in *F. zanthoxyli* proteome. (**A**) Number and classification of CAZymes. (**B**) Number and classification of effectors. (**C**) Venn diagram comparing effectors and CAZymes. (**D**) Systematic phylogenetic analysis of 50 genes shared by CAZymes and effectors in *F. zanthoxyli*. (**E**) Functional categories of 1,143 PHI proteins. (**F**) Systematic phylogenetic analysis of 21 effectors and increased virulence sequences from *F. zanthoxyli* (highlighted in blue) with related sequences from other species

When encountering a potential host plant, phytopathogenic fungi face the challenge of breaking down the plant cell wall, which serves as a barrier against pathogen attack. To overcome this barrier, fungi produce a variety of CAZymes that play crucial roles in degrading the complex network of polysaccharides present in the plant cell wall [34]. In *F. zanthoxyli*, we identified a total of 495 putative CAZyme genes, accounting for 4.37% of its genome (Table S6). These putative CAZyme genes were classified into six classes based on their functions and gene numbers (Fig. 3A). The class with the highest gene count was glycoside hydrolases (GHs) with 241 genes, followed by glycosyl transferases (GTs) with 111 genes, auxiliary activities oxidoreductases (AAs) with 54 genes, carbohydrate esterases (CEs) with 33 genes, carbohydrate-binding modules (CBMs) with 29 genes, and polysaccharide lyases (PLs) with 27 genes. Furthermore, all the identified putative CAZyme genes in *F. zanthoxyli* were further categorized into specific subfamilies within each class. Specifically, there were 82 subfamilies within GHs, 34 subfamilies within GTs, 11 subfamilies within CBMs, 20 subfamilies within AAs, 9 subfamilies within CEs, and 12 subfamilies within PLs. These findings highlight the diverse repertoire of CAZymes in *F. zanthoxyli* that are potentially involved in carbohydrate metabolism and degradation of the plant cell wall.

We also identified a total of 600 sequences in the *F. zanthoxyli* genome that were classified as secretory proteins based on their characteristics as classical secreted proteins. These characteristics include the presence of signal peptides, subcellular localization to the extracellular secretory type, absence of transmembrane domains, and lack of GPI anchors (Table S7). The length of secretory protein ranged from 69 aa to 6548 aa, and the most enriched domains in secretory proteins are mainly related to glycoside hydrolase, peptidases and abhydrolase synthesis.It is worth noting that the number of secreted proteins in *F. zanthoxyli* was relatively lower compared to some other *Fusarium* species, such as *F. oxysporum* f. sp. *lycopersic*i (1119) [35] and *F. solani-melongenae* (1154) [8]. To further identify potential effector proteins in *F. zanthoxyli*, we used EffectorP 3.0, a predictive tool for effectors of plant pathogenic fungi and oomycetes [36]. From the pool of 600 secreted proteins, a total of 217 candidate effector proteins were identified (Fig. 3B; Table S8).The length of effector protein ranged from 69 aa to 1116 aa, and the most enriched domains in secretory proteins are mainly related to glycoside hydrolase and peptidases synthesis, which was similar with the result of secretory proteins. Among these candidates, 156 were predicted to function in the apoplast of the host plant, while 61 were predicted to function in the cytoplasm of the host plant. These candidate effector proteins are likely to play important roles in the pathogen’s ability to invade and colonize host plants by manipulating the plant’s immune response.

Both CAZymes and effector proteins play crucial roles in pathogen infection and colonization of host plants. Interestingly, some CAZymes in plant pathogenic fungi may also function as potential effectors [37, 38], while certain effectors may exhibit CAZyme activities [39]. This dual functionality highlights the versatility and complexity of these proteins in the context of pathogen-host interactions. In our study, we performed a Venn diagram analysis on the predicted 495 CAZymes and 217 effector proteins to identify proteins that possess both CAZyme and effector features (Fig. 3C). The analysis revealed that 50 proteins potentially function as both CAZymes and effectors simultaneously, with 45 of them predicted to be apoplastic effectors (Table S9). A phylogenetic cluster analysis was performed on these 50 dual-functional proteins, revealing that the majority of them belong to GHs with 18 proteins, followed by PLs with 12 proteins (Fig. 3D). This dual functionality of certain proteins further emphasizes the intricate interplay between pathogenic fungi and host plants during infection.

The PHI database is a valuable resource for researchers studying pathogen-host interactions and the molecular mechanisms underlying diseases caused by bacteria, fungi, and oomycetes [40]. In our study, we utilized the PHI database to identify coding genes in *F. zanthoxyli* that are associated with pathogenicity, and effector functions. A total of 1,143 coding genes (10.10% of the *F. zanthoxyli* genome) were identified in the PHI database and classified into eight classes (Fig. 3E; Table S10). Notably, there were 17 genes associated with “increased virulence” and four genes annotated as “effectors”, which have significant implications for the pathogenicity of *F. zanthoxyli* and warrant further investigation. To gain insights into the potential functions of these “increased virulence” and “effector” genes, a BLAST analysis was performed in NCBI, and a phylogenetic tree was constructed to compare the objective genes with the query genes (Fig. 3F). Among the four genes associated with “effectors”, two encoded hypothetical proteins (A05807, A09805), while the other two potentially encoded methionine aminopeptidase 1 (A04850) and RAY38 related protein (A10184), respectively (Table S11). Regarding the genes associated with “increased virulence,” a significant portion of them were predicted to encode hypothetical or uncharacterized proteins, while only a limited number were predicted to have connections with known proteins. These findings provide valuable insights into the potential pathogenic factors of *F. zanthoxyli.* The identification and characterization of these genes contribute to our ongoing research on understanding the interactions between *F. zanthoxyli* and its host plants.

### Gene clusters associated with secondary metabolism in ***F. zanthoxyli***

**Fig. 4:**
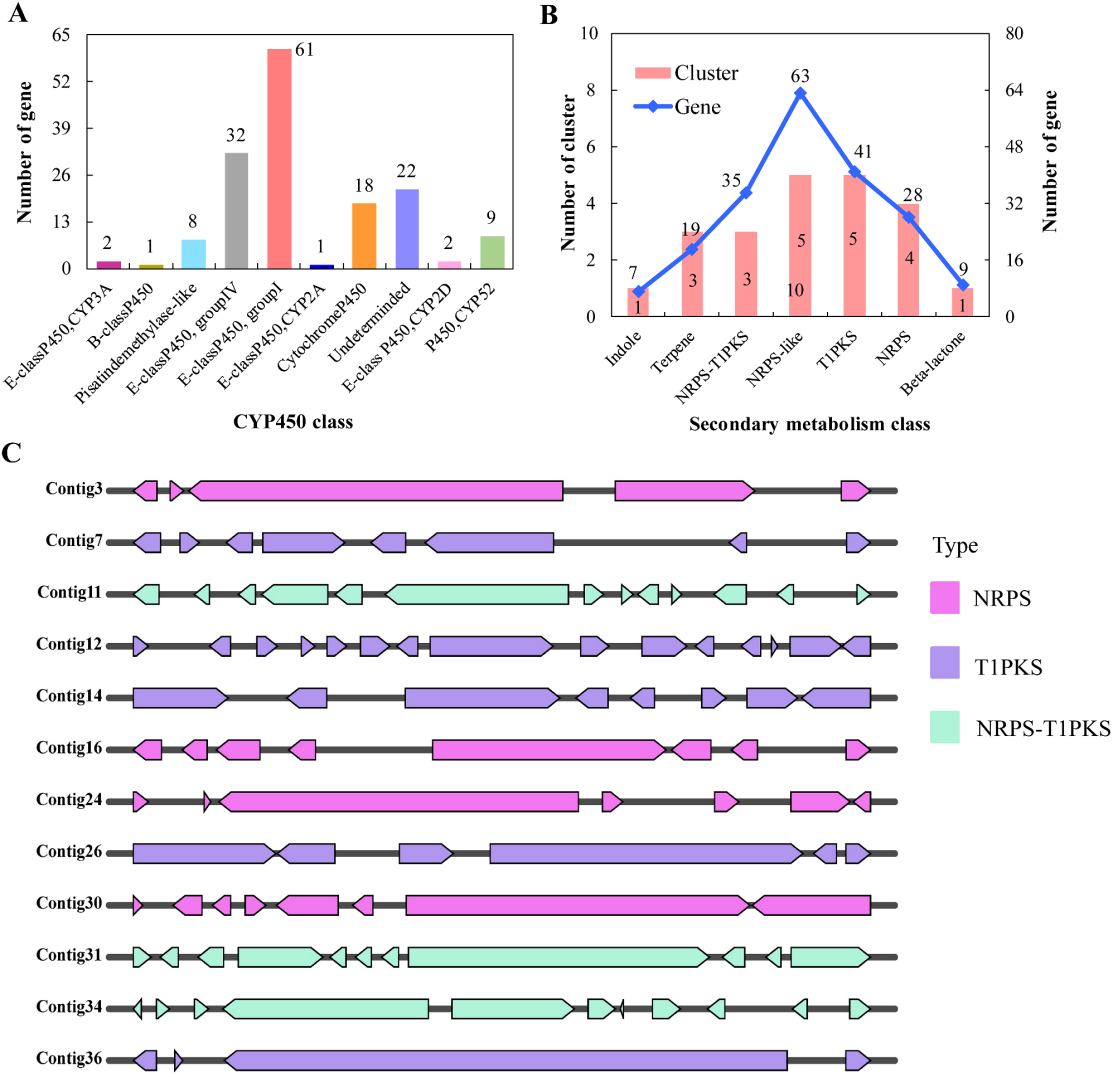
Comprehensive analyses of secondary metabolism-related genes in *F. zanthoxyli*. (**A**) Number and classification of CYP450. (**B**) Number and classification of other secondary metabolism-related genes. (**C**) Arrangement architecture of gene clusters for NRPS, T1PKS and NPRS-T1PKS on contigs

Secondary metabolism plays a crucial role in the pathogenicity of plant pathogen fungi by producing mycotoxins to kill plant cells, such as moniliformin, fumonisin, aflatoxin, etc [41]. The biosynthesis of mycotoxins is believed to be regulated by various genes, including those encoding CYP450, PKS, NRPS, and more. In the case of *F. zanthoxyli*, a total of 156 genes were annotated as CYP450 and categorized into ten classes (Fig. 4A; Table S12). The class with the most annotations was “E-classP450, group I,” followed by “E-classP450, group IV.” Additionally, other genes involved in the secondary metabolism of *F. zanthoxyli* were predicted, including type I PKS (T1PKS), NRPS, hybrid NRPS-T1PKS, RPS-like, indole, terpene, and beta-lactone (Fig. 4B; Table S13). A total of 22 gene clusters containing 202 genes were identified. Notably, there were more gene clusters and gene members associated with the biosynthesis of T1PKS, NRPS, and NRPS-T1PKS. The distribution architecture of these genes and clusters in the genome of *F. zanthoxyli* was further analyzed and constructed (Fig. 4C). Specifically, the genes responsible for NRPS were physically linked together on Contig3, Contig16, Contig24, and Contig30. The T1PKS gene clusters were found on Contig7, Contig14, Contig12, Contig26, and Contig36. The genes encoding NRPS-T1PKS were identified on Contig11, Contig31, and Contig34. These findings highlight the importance of studying the functions of genes associated with the secondary metabolism of *F. zanthoxyli* to further elucidate the biosynthesis mechanisms of mycotoxins.

### Evolutionary Insights of ***F. zanthoxyli*revealed by comparative genomics**

To unravel the palaeohistory of *F. zanthoxyli*, a comparative genomic investigation was conducted on its genome along with 25 other fungal species from eight classes of Ascomycota and Basidiomycota (Table S1). Using Orthofinder, a total of 25,349 orthogroups comprising 330,116 genes were identified, while 42,548 unassigned genes showed no homology in this dataset (Table S14). Among the investigated fungal genomes, 1,430 ortholog families were found to be present in all 26 species, with only 56 species-specific gene families identified in *F. zanthoxyli* (Table S14).

**Fig. 5:**
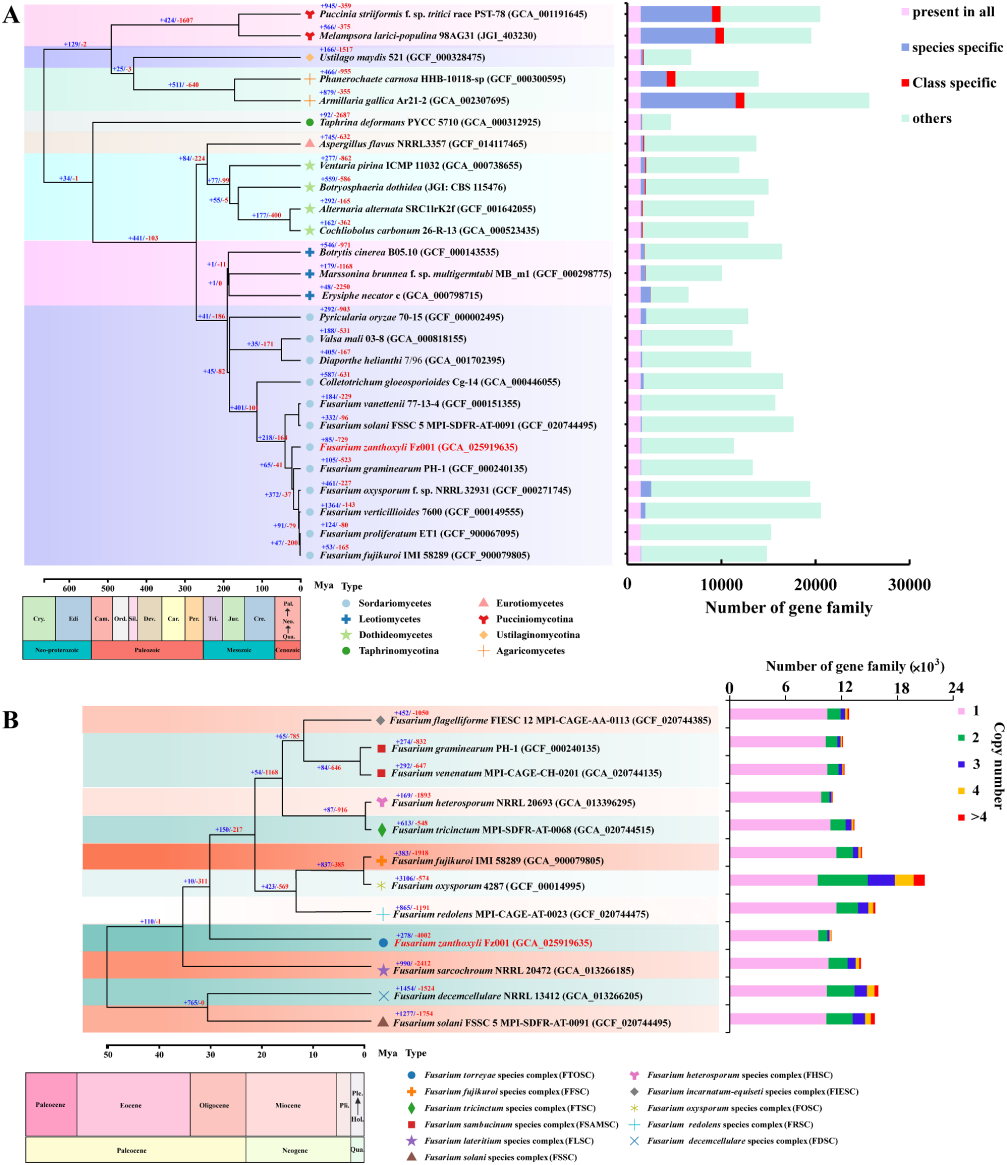
Comparative genomics analysis of *F. zanthoxyli*. (**A**) Phylogenetic placement of *F. zanthoxyli* in the Ascomycota and Basidiomycota phylogenomic tree. Numbers on branches indicate the number of gene gains (+) or losses (−). The estimated divergence times are displayed below the phylogenomic tree. Bar plot on the right of the tree compares gene family construction across the 26 species. Qua.: Quaternary; Neo.: Neogene; Pal.: Paleogene; Cre.: Cretaceous; Jur.: Jurassic; Tri.: Triassic; Per.: Permian; Car.: Carboniferous; Dev.: Devonian; Sil.: Silurian; Ord.: Ordovician; Cam.: Cambrian; Edi.: Ediacaran; Cry.: Cryogenian; Bar plot on the right compares the number of gene family across the 26 species. (**B**) Evolution of *F. zanthoxyli* in the genus of *Fusarium*. Numbers on branches indicate the number of gene gains (+) or losses (−). The estimated divergence times are displayed below the phylogenomic tree. Qua.: Quaternary; Hol.: Holocene; Ple.: Pleistocene; Pli.: Pliocene. Bar plot on the right compares the number of gene copies across the tested *Fusarium* species

Phylogenomic analysis using genes extracted from 332 single-copy orthogroups in the 26 fungal genomes revealed that the *Fusarium* genus shared a common ancestor with the *Colletotrichum* genus approximately 141.1 ~ 88.4 million years ago (MYA). Additionally, apart from *F. vanettenii* and *F. solani*, *F. zanthoxyli* diverged earlier from the genus *Fusarium* at about 27.5 ~ 17.2 MYA compared to the other five *Fusarium* species (*F. graminearum*, *F. oxysporum*, *F. verticillioides*, *F. proliferatum*, and *F. fujikuroi*). Furthermore, expanded and contracted gene families in the genomes of the 26 fungal species were determined using CAFÉ calculation. In comparison to *C. gloeosporioides*, a total of 218 expanded gene families and 164 contracted gene families were respectively identified across all eight checked *Fusarium* species (Fig. 5A). These findings provide valuable insights into the evolutionary history of these fungal taxa, shedding light on their relationships and divergence times.

To further assess the phylogenomic evolution of the *Fusarium* genus, 12 representative species from 11 *Fusarium* species complex were selected to construct a whole-genome phylogram (Table S2). Based on 3,929 single-copy orthologous genes shared among 12 *Fusarium* species, a phylogenomic tree was well generated, revealing the evolutionary relationships across these pathogenic *Fusarium* species (Fig. 5B). Specifically, *F. zanthoxyli* (FTOSC) showed a close affiliation with *F. redolens* (FRSC) and *F. sarcochroum* (FLSC). The divergence of the 12 *Fusarium* species occurred approximately 60.2 ~ 42.9 million years ago (MYA), with *F. decemcellulare* (FDSC) and *F. solani* (FSSC) deviating from the other ten *Fusarium* species during that time period. *F. zanthoxyli* diverged from *F. sarcochroum* around 38.9 ~ 31.7 MYA, while the other eight *Fusarium* species diverged from *F. zanthoxyli* around 34.6 ~ 26.9 MYA. Furthermore, gene copy analyses were performed on all ortholog families of the 12 *Fusarium* species. The majority of gene families in each tested *Fusarium* species consisted of single copies, ranging from 45.16 to 88.64%. In *F. zanthoxyli*, specifically, 87.12% of gene families contained one copy, while 8.81% contained two copies and 4.07% contained more than two copies. The contracted gene families in *F. zanthoxyli* was the most across all the 12 species with the number of 4,002, while a comparatively fewer gene families underwent expansion with the number of 278 (Fig. 5B).

**Fig. 6:**
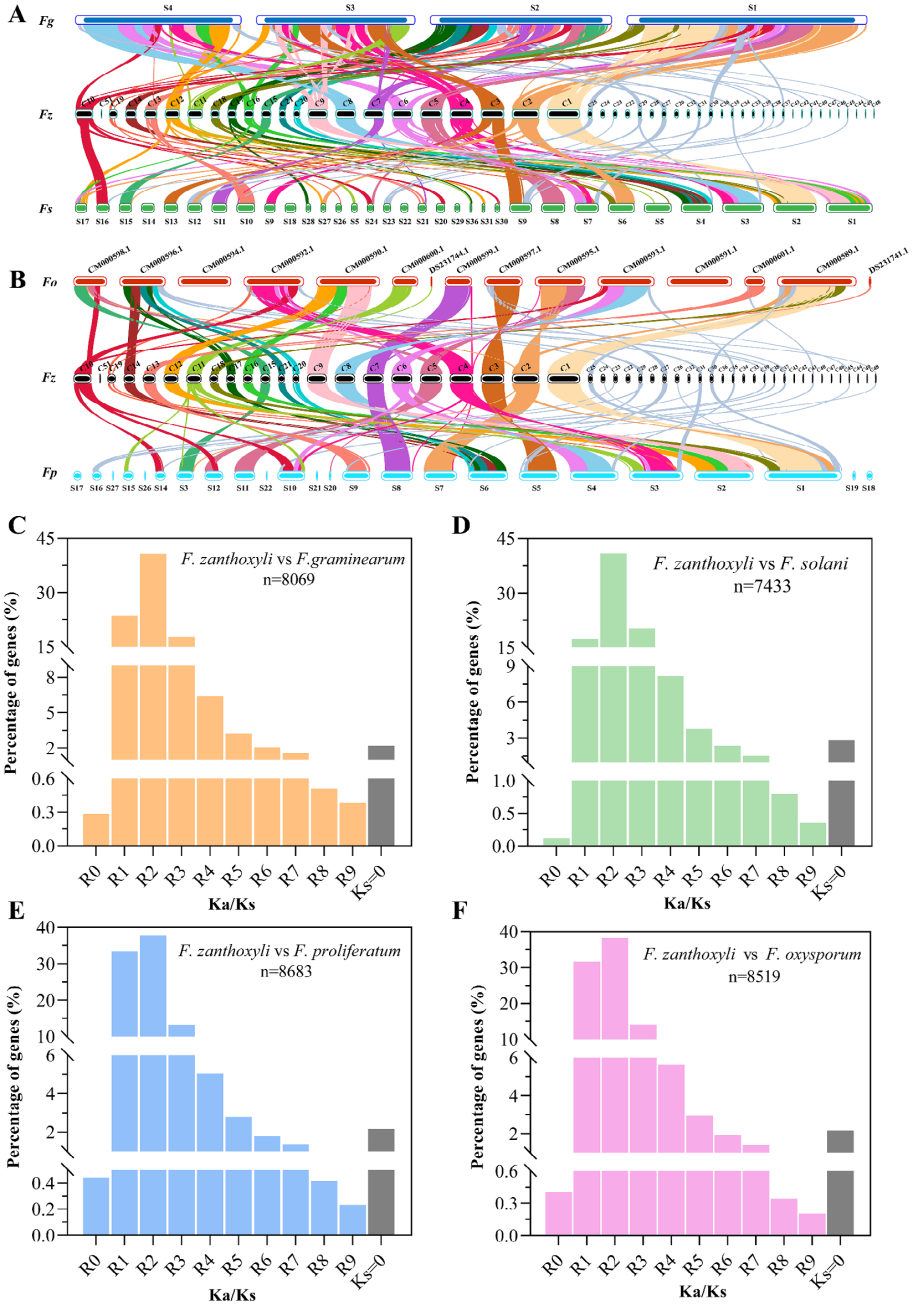
Collinearity and selection pressure analysis of *F. zanthoxyli* genome compared with other *Fusarium* genomes. (**A**-**B**) Genome collinearity shown through synteny blocks. Genome collinearity shown through synteny blocks. Orthologous pairs between *F. zanthoxyli* (Fz) and *F. graminearum* (Fg), *F. solani* (Fs), *F. oxysporum* (Fo), or *F. porotrichioides* (Fp) are connected by different colored lines. (**C**-**F**) Distribution of Ka/Ks ratios for gene pairs between *F. zanthoxyli* and *F. graminearum*, *F. solani*, *F. porotrichioides*, or *F. oxysporum*. The number of all detected gene pairs is indicated by the lowercase letter ‘n’. Abscissa annotation: R0: Ka/Ks = 0.00; R1: 0< Ka/Ks ≤ 0.05; R2: 0.05< Ka/Ks ≤ 0.10; R3: 0.10< Ka/Ks ≤ 0.15; R4: 0.15< Ka/Ks ≤ 0.20; R5: 0.20< Ka/Ks ≤ 0.25; R6: 0.25< Ka/Ks ≤ 0.30; R7: 0.30< Ka/Ks ≤ 0.40; R8: 0.40< Ka/Ks ≤ 0.50; R9: 0.50< Ka/Ks ≤ 0.90

Collinearity analyses were further performed to reveal the distribution or arrangement of homologous genes between *F. zanthoxyli* (FTOSC) and four *Fusarium* species belonging to different species complex, namely *F. graminearum* (FSAMSC), *F. solani* (FSSC), *F. oxysporum* (FOSC) and *F. proliferatum* (FFSC) (Fig. 6; Table S15). The results revealed that several chromosomal segments in *F. zanthoxyli* genome were condensed into one segment in the model species of *F. graminearum*, with some inversions observed. A total of 8,069 orthologous genes in the genome of *F. zanthoxyli* showed collinearity with those of *F. graminearum*, with a collinearity rate of 71.31% (the ratio of collinear orthologous gene to all genes in *F. zanthoxyli*) (Fig. 6A). In contrast, a relatively lower collinearity rate (65.69%) was observed for the analysis between *F. zanthoxyli* and *F. solani* (Fig. 6A). However, relatively higher collinearity rates were calculated for the cases of *F. zanthoxyli*-*F. oxysporum* (78.59%) and *F. zanthoxyli*-*F. proliferatum* (76.73%) (Fig. 6B). These findings suggest that *F. zanthoxyli* is phylogenetically distant from *F. solani*, while it shows a relatively close affiliation with *F. proliferatum .* This observation aligns with the evolutionary analyses previously depicted in Figs. 5 and 6A-B.

To clarify the phylogenetic divergences of *F. zanthoxyli* from the four *Fusarium* species mentioned above, we analyzed the rate of protein evolution for each orthologous gene to uncover the footprints of selection pressure on *F. zanthoxyli*. It can be measured as the rate of nonsynonymous nucleotide substitution per nonsynonymous site (Ka) relative to the underlying neutral mutation rate, which is given by the rate of synonymous substitution per synonymous site (Ks) [42]. The ratios of Ka/Ks for 8,069, 7,433, 8,519, and 8,461 ortholog pairs between *F. zanthoxyli* and *F. graminearum*, *F. solani*, *F. oxysporum*, and *F. proliferatum* were respectively calculated (Table S15). Besides, the distribution of Ka/Ks ratios between *F. zanthoxyli* and these four *Fusarium* species was comprehensively presented (Fig. 6C-F). Overall, the Ka/Ks ratios were less than one, indicating that most orthologs in *F. zanthoxyli* underwent purifying selection and were highly conserved. In the case of *F. zanthoxyli*-*F. graminearum*, we identified 184 genes with Ks values of 0 (Fig. 6C), while for the other three cases, the numbers of genes with Ks values of 0 were 220, 188, and 196 (Fig. 6D-F). These genes with Ks values of 0 were excluded from further analyses due to their Ka/Ks ratio of infinity. The highest average Ka/Ks ratio was found for the case of *F. zanthoxyli-F. solani* with a value of 0.11 (Fig. 6D), while the lowest Ka/Ks ratio was obtained for the case of *F. zanthoxyli-F. proliferatum* with a value of 0.085 (Fig. 6E). The average Ka/Ks ratios for the cases of *F. zanthoxyli-F. graminearum* (Fig. 6C) and *F. zanthoxyli-F. oxysporum* (Fig. 6F) were respectively 0.097 and 0.085. A lower average Ka/Ks ratio implies stronger purifying selection, which may result in a closer affiliation between two species if it is strong enough to decrease Ka and even bring it close to zero. Based on the Ka/Ks ratio analyses of *F. zanthoxyli* with the four *Fusarium* species mentioned above, we conclude that a closer phylogenetic relationship exists between *F. zanthoxyli* and *F. proliferatum*, while a farther phylogenomic evolution was observed between *F. zanthoxyli* and *F. solani.* These findings are consistent with the collinearity analyses presented earlier (Figs. 5 and 6A-B).

### Conserved domain analyses of unique and expanded genes in ***F. zanthoxyli***

**Fig. 7:**
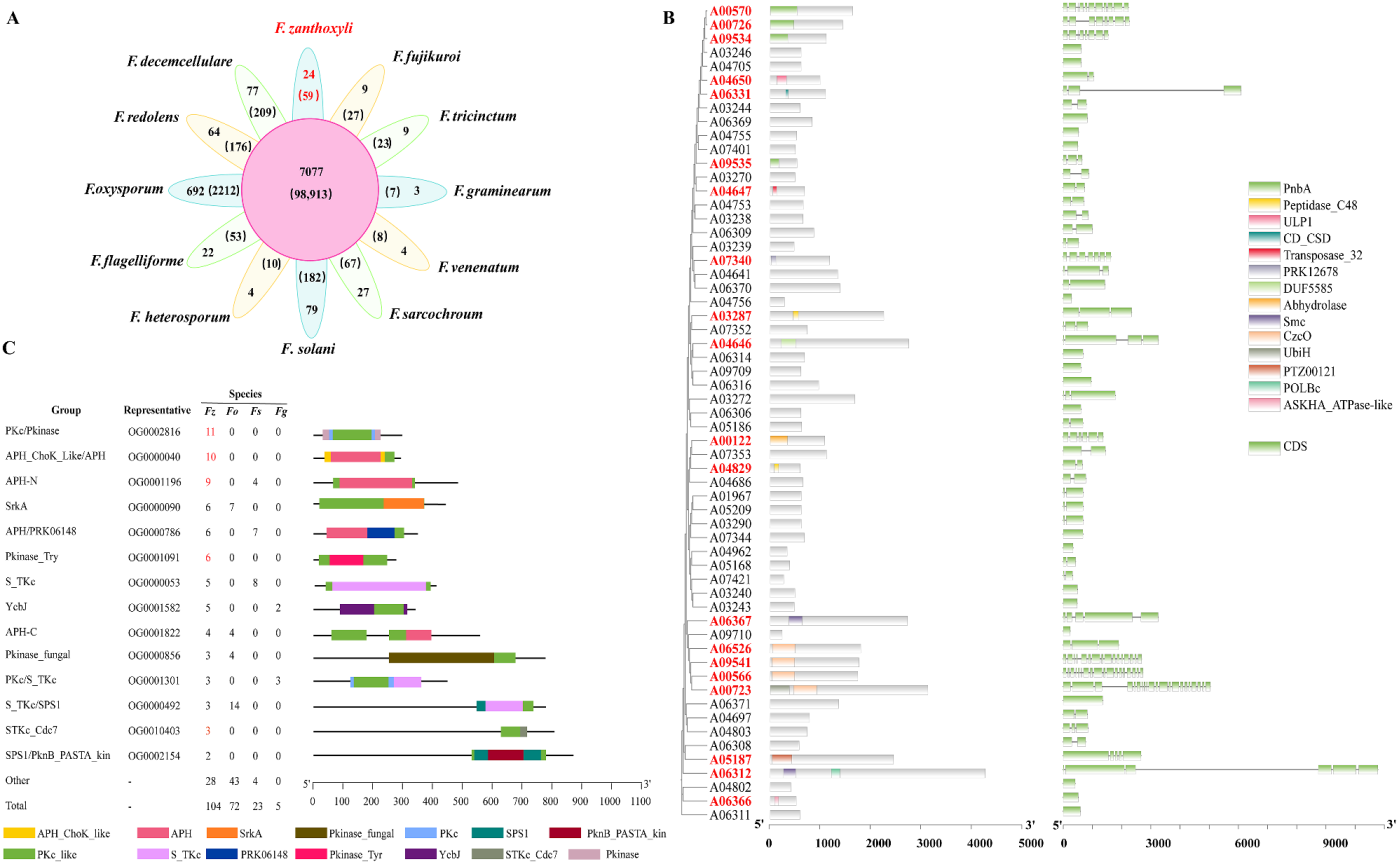
Comparative genomics analysis of *F. zanthoxyli* within the genus *Fusarium*. (**A**) Venn diagram depicting shared and unique ortholog families among 12 *Fusarium* species. Numbers in parentheses indicate gene counts in ortholog families. (**B**) Phylogenetic, conserved domains and exon-intron structures illustrating 57 unique genes of *F. zanthoxyli*. Bootstrap = 5000. (**C**) Comparison of expanded PKc_like proteins in *F. zanthoxyli* (Fz) with *F. oxysporum* (Fo), *F. solani* (Fs), and *F. graminearum* (Fg). Genes exhibiting at least a two-fold higher copy number in *F. zanthoxyli* are highlighted in red

Through analysis of orthologous gene families across 12 *Fusarium* species, a total of 17,799 gene families comprising 177,080 genes were found to be universally shared among all species (Fig. 7A). In the case of *F. zanthoxyli*, these common gene families accounted for 69.74% of its entire gene family repertoire. Interestingly, a mere 24 gene families, encompassing 59 genes, were identified as exclusive to *F. zanthoxyli*, representing a modest 0.24% of its gene families. The 59 unique genes underwent analysis for conserved domains and exon-intron structures (Table S16; Fig. 7B). Specifically, 20 unique genes were predicted to possess conserved domains, with all but two containing only a single domain (A00723 and A06312, which contain two domains each). Notably, four of these genes (A09534, A09535, A00570, and A00726) were predicted to code for PnbA (Carboxylesterase type B), while another four genes (A09541, A00723, A00566, and A06526) were identified as potential encoders of CzcO domain-containing proteins. Three genes (A03287, A04650, and A04829) were predicted contained the domain of ULP1, a pivotal component involved in the SUMO modification process. Two genes (A06312 and A06367) were predicted to contain the domain of Smc, which encompasses ATPases and DNA polymerases involved in chromosome structure maintenance and segregation. This comprehensive analysis of unique genes in the pathogen sheds light on potential virulence factors and molecular pathways crucial for understanding its pathogenicity and devising targeted disease management strategies.

To investigate the potential functions of the expanded genes in *F. zanthoxyli* identified from an evolutionary analysis of the 12 *Fusarium* species (Fig. 5B), we identified a total of 784 expanded genes in 276 orthogroups and subjected them to conserved domain analyses (Tables S17-S18). Notably, among the most highly expanded orthologous gene groups in *F. zanthoxyli* were proteins containing a conserved protein kinase C-like (PKc_like) domain, which were encoded by 104 genes (Table S18). Therefore, we conducted a comprehensive analysis of PKc_like proteins in *F. zanthoxyli* and compared them with those in three typical phytopathogenic *Fusarium* species, namely *F. oxysporum*, *F. solani*, and *F. graminearum* (Fig. 7C). Interestingly, the number of PKc_like proteins in *F. zanthoxyli* was significantly higher than those in the other three *Fusarium* species. Other domains were also identified in the 104 PKc_like proteins, and these were used to categorize the proteins into groups (Fig. 7C). *F. zanthoxyli* contained more copies in most groups than the other three *Fusarium* species, especially the groups of PKc-Pkinase, APH_Chok_like/APH, APH-N, Pkinase_Try, and STKc_Cdc7. In addition, except for Ycbj and PKc/S_TKc, the other groups comprised no copies in the model species *F. graminearum* but were expanded significantly in *F. zanthoxyli* (Fig. 7C).

## Discussion

Fungi have a notable impact on human welfare as they can destroy valuable crops as devastating pathogens or producers of mycotoxins [34]. However, the limited understanding of the molecular mechanisms of fungal pathogenesis has hindered the precise prevention and control of plant diseases. The application of genomic technologies holds great promise in revealing the pathogenicity factors of pathogenic fungi and improving our understanding of host-pathogen interactions. The absence of a relatively high-quality genome of *F. zanthoxyli*, the pathogen responsible for stem canker of *Z. bungeanum*, has hindered research on its interaction with *Z. bungeanum* and impeded the development of precise control strategies for stem canker. Although a 40.9 Mb genome assembly for *F. zanthoxyli* NRRL 66,285 is publicly available in GenBank (GCA_013623745.1), it lacks gene annotations and contains an extremely large scaffold number (2,368) and a short N50 scaffold length (0.053 Mb). In the present study, we sequenced, assembled, and annotated the genome of *F. zanthoxyli* Fz001, resulting in a relatively high-quality genome with a size of 43.39 Mb containing 55 contigs with a contig N50 value of 2.09 Mb (Table 1). Compared with the model strain of the model *Fusarium* species, *F. graminearum* PH-1, the causative agent of wheat scab, which has a genome size of 36.5 Mb. *F. zanthoxyli* has a larger genome. The importance of genome size variations in phytopathogenic fungi lies in its impact on pathogenicity and adaptation. Generally, larger genomes may contain a higher number of genes involved in pathogenicity-related processes such as host recognition, toxin production, and immune evasion [43, 44]. Understanding the reasons for genome size variations among phytopathogenic fungi will provide insights into their evolutionary strategies and may aid in the development of effective strategies for disease management.

Understanding the pathogenic factors of plant pathogenic fungi is of utmost importance in plant pathology research. By identifying and characterizing specific pathogenic factors and understanding how they contribute to infection, researchers can develop novel approaches such as genetic engineering, fungicides, or biocontrol agents to disrupt or inhibit their activity [45]. Studying the genome of plant pathogenic fungi is essential for understanding the genetic basis of their pathogenicity, unraveling host-pathogen interactions, and identifying pathogenic factors. Based on the acquisition of the genome of a plant pathogen fungus, its potential pathogenic factors, such as CAZymes, effectors and secondary metabolite synthases, can be preliminary predicted using bioinformatics technology. In our study, we predicted 495 CAZymes coding genes in the genome of *F. zanthoxyli*, a hemibiotrophic pathogen (Fig. 3A; Table S6), which was dramatically larger than those in biotrophic phytopathogenic fungi, such as *Melampsora laricis-populina* [46], *Blumeria graminis* [47] and *Ustilago maydis* [48], etc. This finding is consistent with the previous viewpoint that hemibiotrophic phytopathogenic fungi have more CAZymes than biotrophic pathogens [49]. Hemibiotrophic fungi often cause necrotic symptoms in host tissues during the later stages of infection. To facilitate tissue degradation and nutrient release, they produce a higher number of CAZymes involved in cell wall degradation such as cellulases and pectinases [50]. Besides, hemibiotrophic fungi have a broader ecological niche and can switch between biotrophic and necrotrophic lifestyles during their infection cycle [51]. This versatility requires a larger repertoire of CAZymes to adapt to different nutritional requirements in different stages of infection [52].

Another type of pathogenic factors, effectors, were further predicted in the genome of *F. zanthoxyli*. A total of 217 effectors, including both apoplastic and cytoplasmic effector proteins, were obtained in *F. zanthoxyli* (Fig. 3B; Table S8). Identifying effectors and understanding their functions and targets are essential for understanding virulence mechanisms, host-pathogen interactions, developing resistant crop varieties, diagnostic tools, and exploring biotechnological applications [53]. For example, Wang et al. [54] identified the key susceptible gene *TaPsIPK1* in the host plant through the pathogenic effector protein PsSpg1 of wheat stripe rust fungus, opening up a new avenue for molecular breeding of disease-resistant wheat using modern gene editing techniques. The CYP450 enzymes play crucial roles in virulence, adaptation to host environments, evasion of host immune responses, and mycotoxin formation in many plant pathogens [55], such as *V. dahliae* [56], *Nectria haematococca* [57], and *F. graminearum* [58]. Further analysis of *F. zanthoxyli* effectors revealed that 50 sequences also had CAZymes characteristics and most of them belong to the class of GHs (Fig. 3C-D; Table S9). Secreted GHs of plant-associated fungi and oomycetes have been reported to act as effectors to promote microbial host colonization or activating the plant immune system [59, 60]. In addition, *Fusarium* can produce abundant secondary metabolites, including polyketide and non-ribosomal peptide, which can directly damage the cells and tissues of host plants, breach plant defense barriers, and even suppress the immune system response, thereby promoting the development of disease [61]. The biosynthesis of these metabolites is often regulated by numerous genes such as *CYP450*, *PKS*, *NRPS*, and others [62]. For instance, the biosynthesis of Fusarin C, a polyketide specific to *Fusarium*, was reported to be regulated by a heterozygous gene *FUSS* encoding NRPS-T1PKS in *F. moniliforme* and *F. venenatum* [63]. These findings on the potential pathogenic factors will contribute to our future researches on the interactions of *F. zanhtoxyli* and *Z. bungeanum*.

Comparative genomics has been widely applied in plant pathology to identify pathogens, unveil their evolutionary links, estimate their divergence times for groups originating from a shared ancestor, and anticipates novel pathogenic genes, etc [64]. In the present research, comparative genomics analyses revealed that the *Fusarium* genus shared a common ancestor with the *Colletotrichum* genus approximately before 141.1 ~ 88.4 MYA, suggesting a close molecular affiliation between them (Fig. 5A). Both genera the *Fusarium* and *Colletotrichum* belong to the hemibiotrophic trophic strategy, with a broad host range, capable of parasitizing various parts of plants, causing diverse symptoms, and widely distributed in nature. Comparative genomics analysis revealed insights into the size of the GH32 family between *Fusarium* and *Colletotrichum*, reflecting adaptations of GH32 to different ecological niches or substrate availabilities [65]. This adaptability is also evident in *Valsa mali*, the pathogen causing apple canker, as revealed by comparative genomics analysis, reflecting its strategies to cope with nutrient limitations and low pH environment in the bark, as well as its specialization in pectin degradation while being restricted by cellulose and lignin degradation [66]. Thus, the genome of *F. zanthoxyli*, belonging to the complex FTOSC, was integrated with eleven *Fusarium* genomes belonging to ten species complexes to construct a comprehensive super data tree (Fig. 5B). Although the phylogeny of *F. zanthoxyli* has also been reported in previous studies [5], no evolutionary divergence time was calculated, resulting in a lack of information on its evolutionary divergence. In this study, all 12 *Fusarium* species shared a common ancestor approximately 60.2 ~ 42.9 MYA, while *F. zanthoxyli* started to diverge in the middle Oligocene period approximately 34.6 ~ 26.9 MYA. By referring to the evolutionary divergence time of *Z. bungeanum*, the host of *F. zanthoxyli*, which diverged in the middle Oligocene period approximately 35.3 MYA [3], we speculate that *F. zanthoxyli* might have started parasitizing *Z. bungeanum* during the middle Oligocene period.

A stable plant disease is the results of the long-term co-evolution between a pathogen and its host plants. The evolutionary adaptation, functional diversification, and parasitic adaptability of phytopathogens might be attributed to the expansion and contraction of genes in their genomes [67]. In this study, we identified 276 expanded gene families and 3,014 contracted gene families compared to the aforementioned 11 *Fusarium* species, which is the first report on the gene expansion and contraction of the typical *Fusarium* plant pathogens belonging to different species complexes on genomic level (Fig. 5B). The events of gene expansion and contraction in *F. zanthoxyli*, a fungal pathogen that specifically parasitizes *Z. bungeanum*, are likely closely related to its specialized parasitic adaptability to this particular host. Indeed, variations in gene expansion and contraction observed among different fungal pathogens have been reported to attribute to their evolutionary divergence and adaptation to different external environments [68]. Factors such as host range, ecological niche, and environmental conditions can influence the selection pressures acting on these fungal pathogens, resulting in different patterns of gene expansion and contraction [69]. The differences in host range, ecological niche, and environmental conditions among the aforementioned 11 query *Fusarium* species exist indeed. For example, *F. graminearum* primarily infects cereal crops to cause scab [70], *F. oxysporum* primarily colonizes the xylem of plants to cause wilt [71], while *F. solani* mainly inhabits in soil to cause root rot [72]. These differences highlight the role of host specialization and environmental adaptation in shaping the genomic architecture of *Fusarium* plant pathogens. By studying these variations, it will be helpful to gain insights into the genetic basis of host specificity, pathogenicity, and ecological adaptation of *F. zanthoxyli*.

The exclusive genes identified in a specific plant pathogenic fungus hold pivotal significance in comprehending the molecular mechanisms underpinning pathogen infections, devising diagnostic tools, formulating targeted control strategies, and unraveling the pathogen’s evolutionary dynamics [73]. In this study, we conducted a comparative genomics analysis of 12 *Fusarium* species, resulting in the identification of 24 gene families encompassing a total of 57 genes, that were found to be unique to *F. zanthoxyli* (Fig. 7A). Abhydrolase has been recognized as a crucial virulent factor in certain pathogenic fungi, such as *F. graminearum* [74] and *Sclerotinia sclerotiorum* [75]. Meanwhile, CzcO domain-containing proteins have been shown to play important roles in fungal development and plant infection in *M. oryzae* [76] and *F. fujikuroi* [77]. The role of Ulp1 in yeast involves regulating the protein modification state within cells by removing SUMO/Smt3 modifications and processing precursor SUMO [78]. Carboxylesterases, which include the PnbA variant, facilitate the hydrolysis of carboxylesters into alcohol and carboxylic acid, thereby serving as a pivotal defense mechanism against ester-containing xenobiotics with a wide range of substrate specificity [79]. The functions of proteins harboring SMC_prok_B or POLBc domains in the pathogenicity of plant fungi remain largely unexplored.

In the context of the plant-pathogen co-evolutionary arms race, the ongoing development of host resistance imposes a persistent selective pressure favoring the retention and expansion of genes associated with virulence [67]. The present study identified 784 expanded genes in 276 orthogroups in *F. zanthoxyli*, with PKc_like and APH_ChoK_Like family being the most expanded gene family (Fig. 7C; Tables S17-S18). The PKc_like proteins have been reported to play important roles in the growth and pathogenicity of various plant pathogens. For example, *Mps1p*, a PKc gene in *M. oryzae*, is a stealth factor that conceals the cell wall of infectious hyphae and prevents recognition by hosts [80]. Previous studies have shown that three *AaCaMKs* genes encoding PKc_like kinases in *A. alternata* regulate infection structure differentiation and pathogenicity [81]. The APH_ChoK_Like family employs ATP-mediated phosphate transfer to chemically modify and inactivate aminoglycoside antibiotics such as streptomycin and kanamycin [82]. This family also encompasses catalytic domains of other kinases, such as typical serine/threonine/tyrosine protein kinases (PKs) [83]. Additionally, we have found that the expanded genes in F. zanthoxyli have been extensively studied and reported to function in numerous pathogenic conditions. Tyrosinase and PKS are involved in the synthesis of melanin, a virulence factor that contributes to fungal survival, adaptability, and infection in many phytopathogenic fungi, including *Bipolaris sorokiniana* [84], *Verticillium dahliae* [85], *Lasiodiplodia gilanensis* [86], and *Alternaria alternata* [87]. ABC transporters are known to be involved in a pathogen’s resistance to cytotoxic compounds or fungicides, enabling survival in unfavorable environments [88]. Currently, only small number of ABC transporter genes have been functionally analyzed in phytopathogenic fungal species, namely, *M. oryzae* [89, 90], and *F. graminearum* [91]. Therefore, further investigation of these unique and expanded genes may provide insights into the co-evolutionary dynamics between *F. zanthoxyli* and *Z. bungeanum.*

## Conclusion

In this study, we sequenced and assembled a relatively high-quality genome of *F. zanthoxyli* and comprehensively predicted potential pathogenicity-related genes, including CAZymes, effectors, and secondary metabolite synthases. Comparative genomics was employed to unravel the phylogenomic evolutionary relationships, divergence times, and gene expansion/contraction events in *F. zanthoxyli*. *Fusarium* and *Colletotrichum* diverged from a shared ancestor approximately 141.1 ~ 88.4 MYA, and *F. zanthoxyli* originated approximately 34.6 ~ 26.9 MYA. Finally, utilizing conserved domain prediction, the results revealed that among the 59 unique genes, the most enriched domains were PnbA and ULP1. Among the 783 expanded genes, the most enriched domains were PKc_like kinases and those of the APH_ChoK_Like family. These findings provide valuable insights into the molecular regulatory mechanisms underlying the adaptation of *F. zanthoxyli* in *Z. bungeanum* stems, and will hold great promise for the development of precise control measures for *Z. bungeanum* stem canker.

### Electronic supplementary material

Below is the link to the electronic supplementary material.

Supplementary Material 1

Supplementary Material 2

## Data Availability

The authors state that all data necessary for confirming the conclusions presented in the article are presented fully within the article and supplemental materials. The raw sequencing data for the genome and the assembly reported in this paper is associated with NCBI BioProject: PRJNA892234 and BioSample: SAMN31372358 within the GenBank.
