# Supplementary material for: Genome sequencing and comparative genomics reveal insights into pathogenicity and evolution of Fusarium zanthoxyli, the causal agent of stem canker in prickly ash
Source: BMC Genomics. 2024 May 21;25:502. doi: 10.1186/s12864-024-10424-w (PMC11110190; doi:10.1186/s12864-024-10424-w)
Supplement: Supplementary file 1 — Supplementary Material 1 [file 12864_2024_10424_MOESM1_ESM.docx]

**Supplementary Figures**

**Title: Genome sequencing and comparative genomics reveal insights into pathogenicity and evolution of *Fusarium zanthoxyli*, the causal agent of stem canker in prickly ash**

Zhao Ruan^a, 1^, Jiahui Jiao^a, 1^, Junchi Zhao^a^, Jiaxue Liu^a^, Chaoqiong Liang^b^, Xia Yang^a^, Yan Sun^a^, Guanghui Tang^a^, Peiqin Li^a*^

**Affiliations:**

^a^ Key Laboratory of National Forestry and Grassland Administration on Management of Western Forest Bio-Disaster, College of Forestry, Northwest A&F University, Yangling, Shaanxi 712100, People’s Republic of China

^b^ Shaanxi Academy of Forestry, Xi’an, Shaanxi, 710082, People’s Republic of China

^*^ Corresponding Author: [lipq@nwsuaf.edu.cn](mailto:lipq@nwsuaf.edu.cn)

^1^ These authors contributed equally to this work.

Zhao Ruan: [ruanzhao1826@163.com](mailto:ruanzhao1826@163.com); Jiahui Jiao: [jjh665605@163.com](mailto:jjh665605@163.com); Junchi Zhao: [catherinezjc@163.com](mailto:catherinezjc@163.com); Jiaxue Liu: [liujx970820@163.com](mailto:liujx970820@163.com); Chaoqiong Liang: [lcq19880305@126.com](mailto:lcq19880305@126.com); Xia Yang: [yangxia12390@126.com](mailto:yangxia12390@126.com); Yan Sun: [sunyan2021055591@163.com](mailto:sunyan2021055591@163.com); Guanghui Tang: [tanggh@nwsuaf.edu.cn](mailto:tanggh@nwsuaf.edu.cn); Peiqin Li: [lipq@nwsuaf.edu.cn](mailto:lipq@nwsuaf.edu.cn)


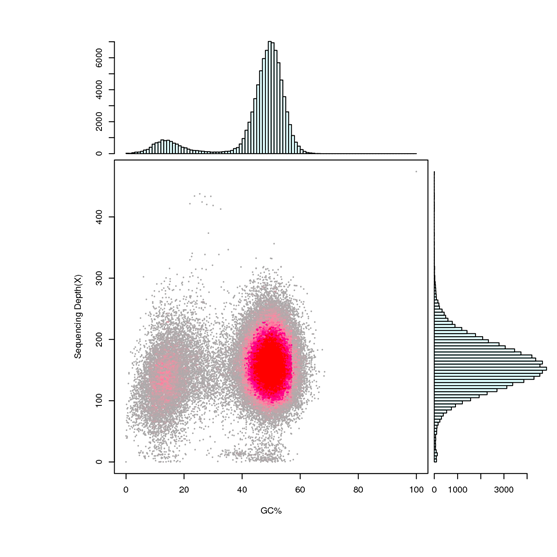
 **Figure S1:** Correlation Analysis and Statistical Plot of Sample GC Content and Sequencing Depth (Depth); Description: The x-axis represents the GC content, the y-axis represents the sequencing depth, and the distribution of sequencing depth is shown on the right, while the distribution of GC content is displayed on the top.


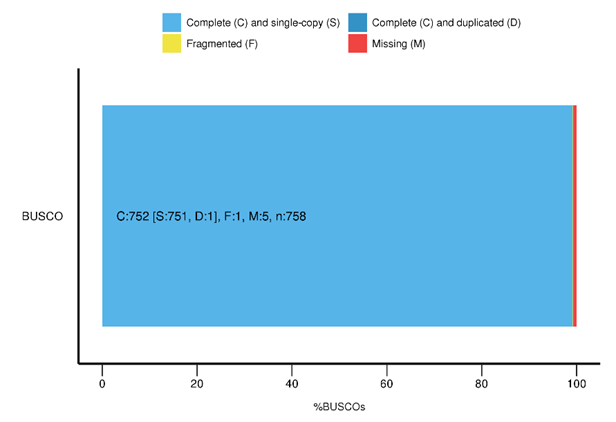


**Figure S2:** BUSCO assessment results


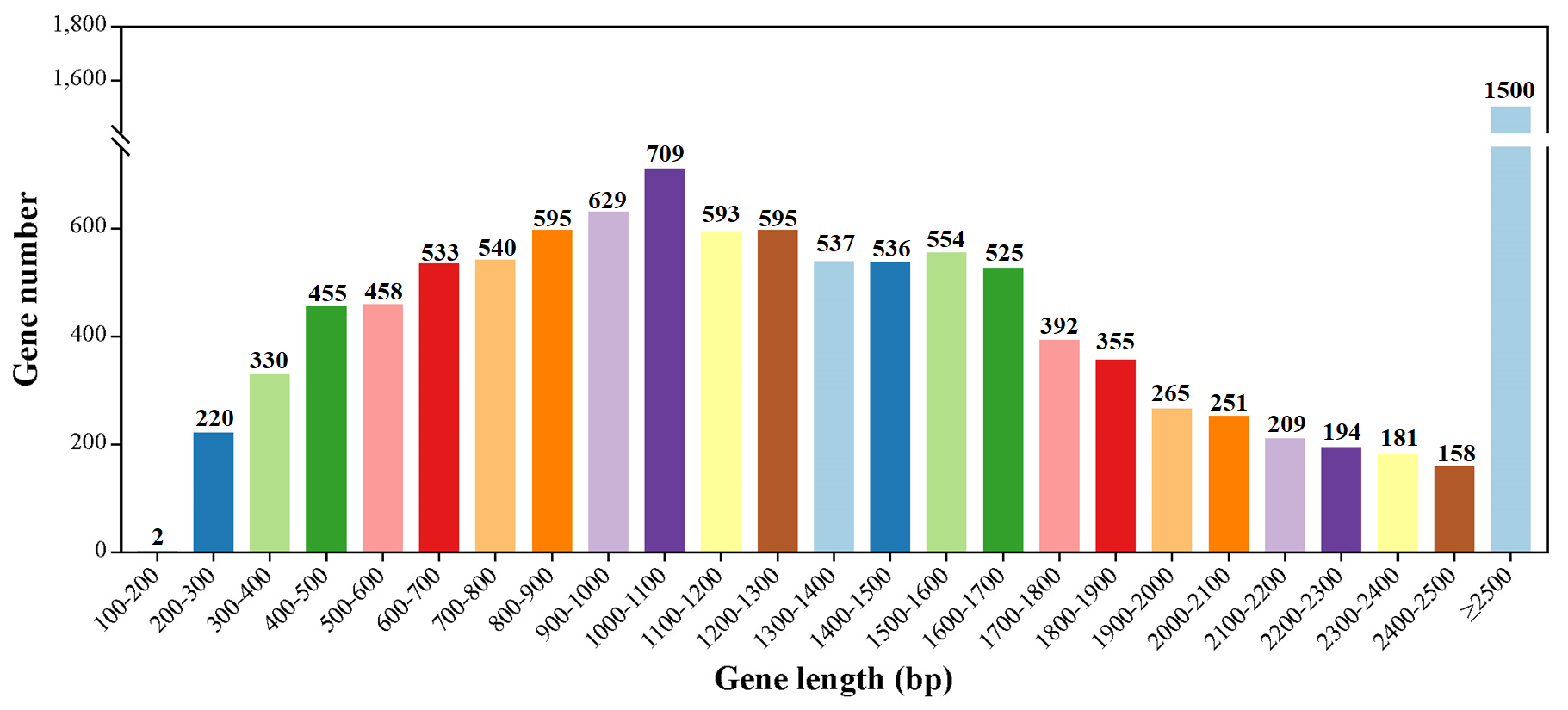


**Figure S3:** Gene length distribution and annotations overview of the *F. zanthoxyli* genome.


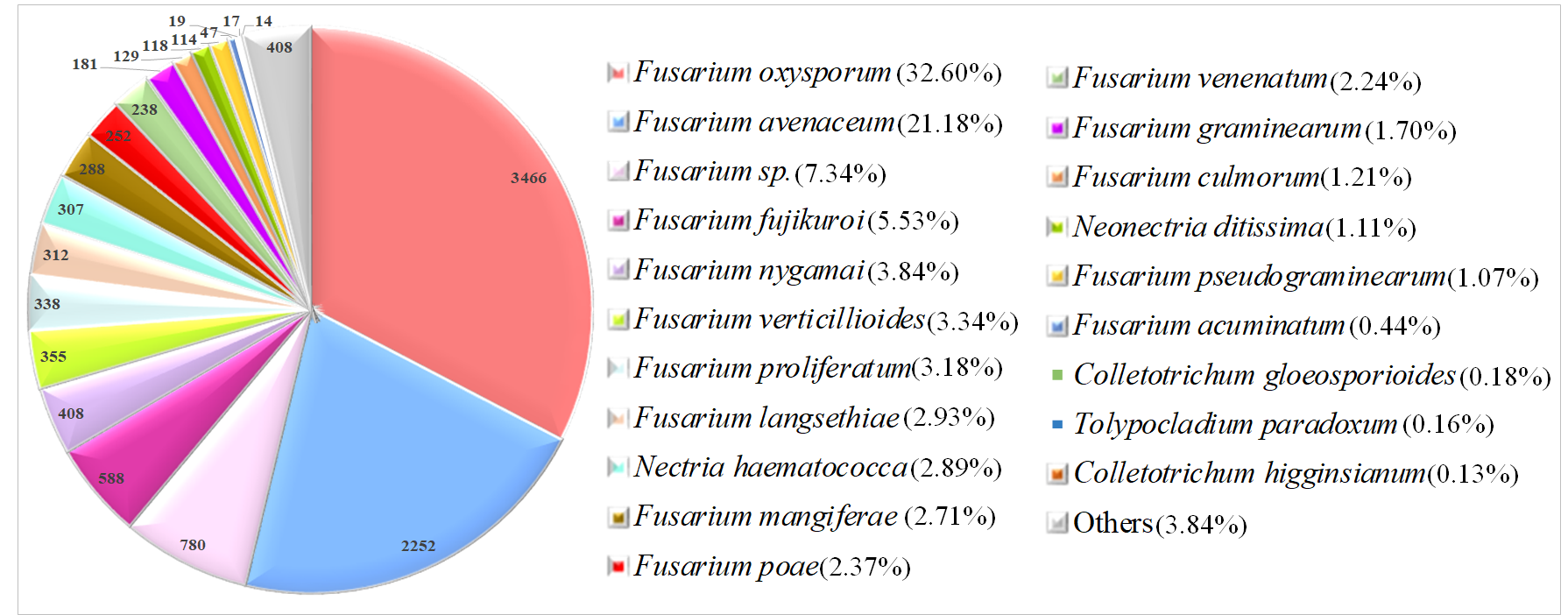


**Figure S4:** Distribution of annotated homologous species in the NR database for *F. zanthoxyli* genome.

**Figure S5:** Gene function annotation of *F. zanthoxyli* in KEGG databases.


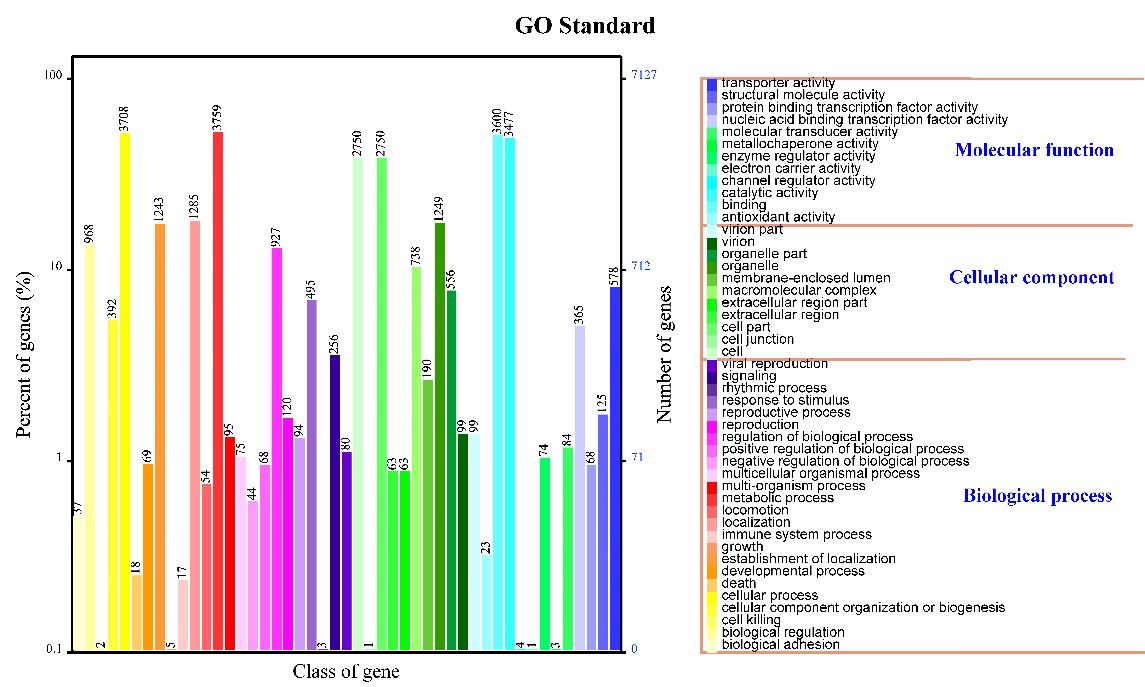


**Figure S6:** GO functional annotation for the genome of *F. zanthoxyli*.


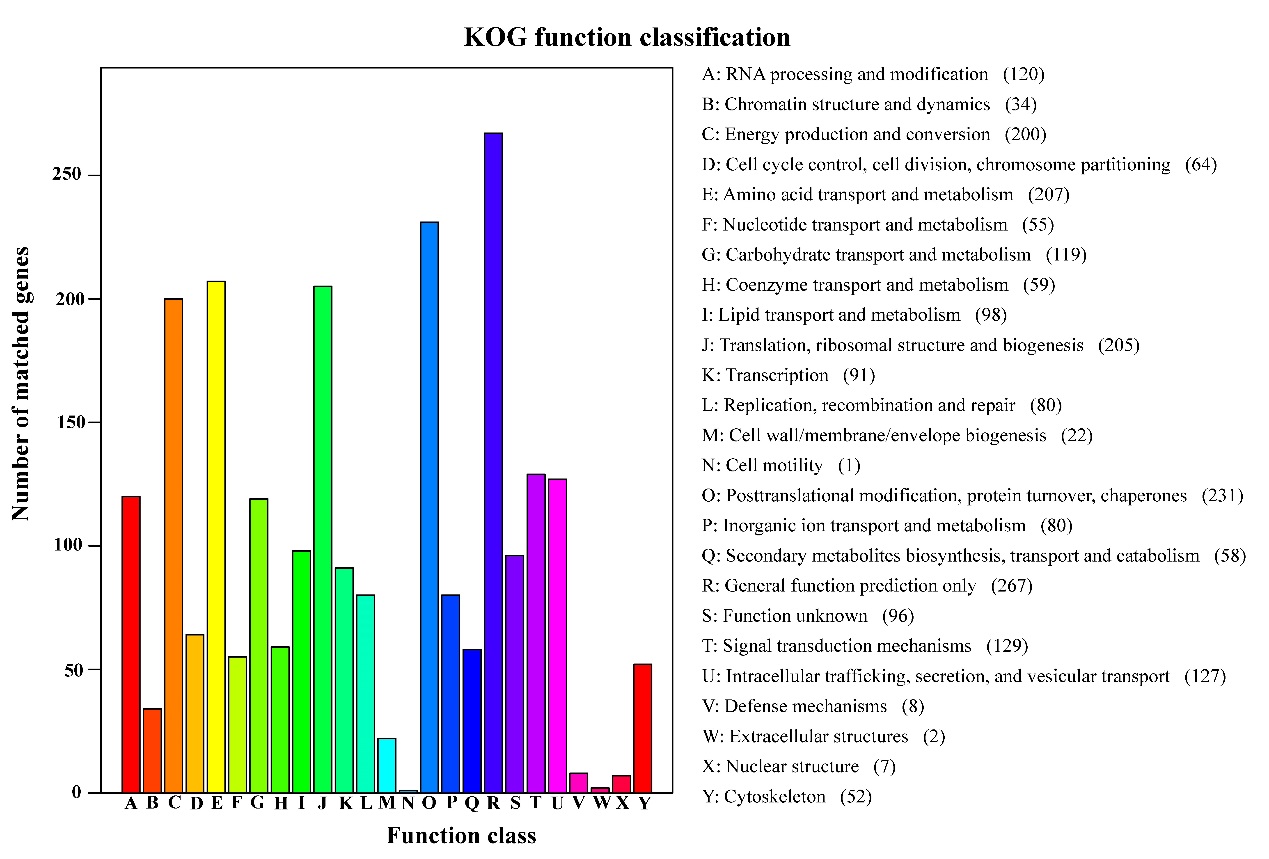


**Figure S7:** Function classification of *F. zanthoxyli* based on KOG database annotation.


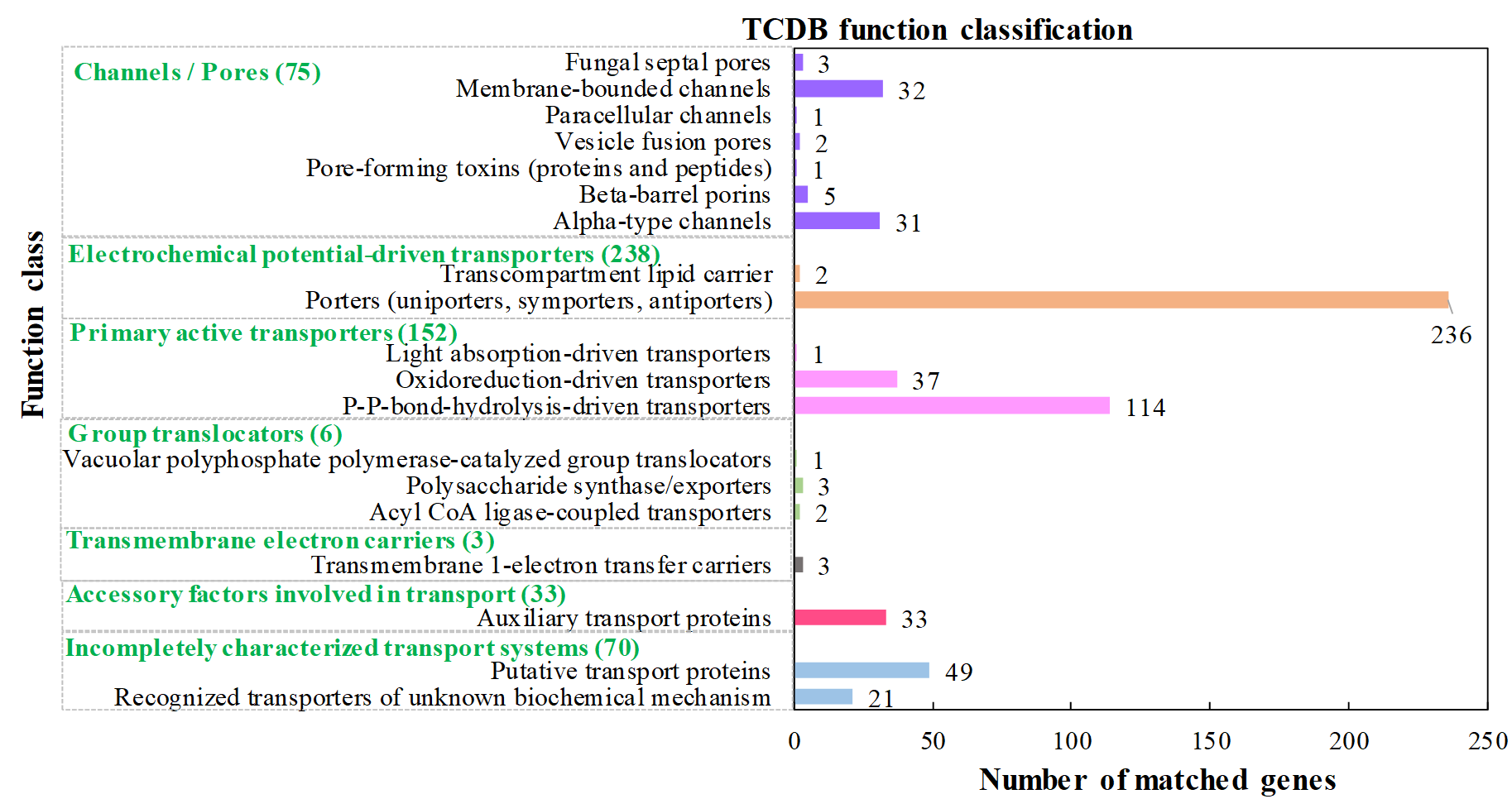


**Figure S8:** Gene function annotation of *F. zanthoxyli* in TCDB databases
